# Duplex-RT-PCR assay for the simultaneous detection and discrimination of Brome mosaic virus and Cocksfoot mottle virus in cereal plants

**DOI:** 10.1007/s11033-021-06485-9

**Published:** 2021-06-16

**Authors:** Katarzyna Trzmiel

**Affiliations:** Department of Virology and Bacteriology, Institute of Plant Protection-National Research Institute (IPP-NRI), ul. Wł. Węgorka 20, 60-318 Poznań, Poland

**Keywords:** BMV, CfMV, Diagnostics, Duplex-RT-PCR, Co-infection, Cereal plants

## Abstract

Brome mosaic virus (BMV) and cocksfoot mottle virus (CfMV) are pathogens of grass species including all economically important cereals. Both viruses have been identified in Poland therefore they create a potential risk to cereal crops. In this study, a duplex—reverse transcription—polymerase chain reaction (duplex-RT-PCR) was developed and optimized for simultaneous detection and differentiation of BMV and CfMV as well as for confirmation of their co-infection. Selected primers CfMVdiag-F/CfMVdiag-R and BMV2-F/BMV2-R amplified 390 bp and 798 bp RT-PCR products within coat protein (CP) region of CfMV and replicase gene of BMV, respectively. Duplex-RT-PCR was successfully applied for the detection of CfMV-P1 and different Polish BMV isolates. Moreover, one sample was found to be co-infected with BMV-ML1 and CfMV-ML1 isolates. The specificity of generated RT-PCR products was verified by sequencing. Duplex-RT-PCR, like conventional RT-PCR, was able to detect two viruses occurring in plant tissues in very low concentration (as low as 4.5 pg/µL of total RNA). In contrast to existing methods, newly developed technique offers a significant time and cost-saving advantage. In conclusion, duplex-RT-PCR is a useful tool which can be implemented by phytosanitary services to rapid detection and differentiation of BMV and CfMV.

## Introduction

Brome mosaic virus (BMV) is typical member of the Bromovirus genus in the *Bromoviridae* family with tripartite genome. RNA1 and RNA2 are associated with virus replication and encode 1a and 2a proteins, respectively while 5′ part of RNA3 encode movement protein (MP). Coding sequence of coat protein (CP) is located on subgenomic RNA4 [1]. The virus is widely distributed and viral infections have been confirmed in North America, South Africa and Europe [2]. Due to the broad range of its hosts [3] to which the virus can be easily transmitted mechanically or by insect vectors (mainly beetles but also dagger nematodes and aphids) [4], as well as the presence of infectious BMV particles in irrigation runoff from cereal commercial fields [5] the pathogen persistence in the environment can be assured. BMV is considered as a model for RNA virus biology [6]. However, despite a lot of information on its basic biology, virus harmfulness information and its impact on crop production is very limited. Generally, BMV has not been considered as a major pathogen so far [7]. Nonetheless, the studies performed by Hodge et al. [2] showed a significant, reaching up to 61%, reduction in grain yield harvested from wheat plants infected with BMV-OH. The first information about BMV in Poland comes from 1999 [8]. The results of studies, conducted in 2012–2020, revealed BMV infections of various crop species, wild plants as well as presence of infectious virus particles in water samples taken from drainage ditches and canals surrounding commercial fields in Wielkopolska, Dolny Śląsk and Małopolska regions in Poland [5, 8].

The second virus, a new potential threat for cereal crops, is cocksfoot mottle virus (CfMV). This is single stranded RNA virus belonging to the Sobemovirus genus in the *Solemoviridae* family. The virus has positive single-stranded polycistronic RNA genome of 4082 nt in size, encapsidated in a spherical particles of about 30 nm in diameter [9]. The 5′ terminus of the RNA has a genome-linked protein (VPg) and the 3′ end lacks a poly(A) tail [10]. The virus was first reported in Great Britain and then in other European countries (Denmark, Norway, France, Germany), Asia (Russia and Japan), North America (Canada, USA) and New Zealand [9]. In Poland CfMV was detected for the first time in 2016 [11]. The virus is easily mechanically transmitted with the sap of infected plants and by vectors—beetles (O*ulema melanopus* and *O. gallaeciana*) in non-persistent manner. CfMV induces lethal, severe or mild leaf mottle and stunting of infected plants [12]. It has relatively narrow host range. In nature, CfMV mainly infected and induced yield losses of *Dactylis glomerata* but it was revealed also in wheat and it could be artificial transmitted to other cereals [12, 13]. As in the case of BMV, also CfMV can become a new potential threat to wheat crops [13]. Since both viruses have morphologically identical spherical virions, are transmitted by the same vectors, and induce similar symptoms on infected plants, proper diagnostics of these viruses can be problematic. To date, detection of BMV and CfMV is based on biological tests, enzyme-linked immunosorbent assays (ELISA) or reverse-transcription-polymerase chain reactions (RT-PCR) [2, 5, 8, 11–19]. A multiplex-reverse transcription-polymerase chain reaction (multiplex-RT-PCR) is the promising alternative for above mentioned techniques which has been developed for the rapid and efficient detection of many RNA viruses in one reaction [5, 20, 21]. For this purpose, in this study a duplex-RT-PCR was developed and optimized for simultaneous detection, and differentiation of BMV and CfMV and verification of their co-infection in cereal plants.

## Material and methods

The studies were conducted with total RNA isolated from barley cv. Bażant infected with several BMV and CfMV-P1 isolates collected in 2013–2020 from different locations in Wielkopolska, Dolny Śląsk and Małopolska regions of Poland (Table 1). The plants were mechanically inoculated with tissue from symptomatic leaves ground with 0.05 M potassium phosphate buffer (pH 7.0) containing carborundum and kept in insect proof glasshouse, in standard conditions (16 h of light and 8 h of darkness at 23 °C). RNA from healthy barley cv. Bażant plants was used as a negative control. Total RNA was extracted using the Total RNA Purification Kit Novazym (Novazym, Poznań, Poland) according to the manufacturer’s protocol approximately 14 days after inoculation. The RNA concentration and quality were estimated using a NanoDrop 2000 spectrophotometer (Nanodrop Technologies, Delawere, USA). For BMV detection BMV2-F/BMV2-R primer pair [17] corresponding to genome sequence coding fragment of RNA-dependent RNA polymerase (RdRp) have been chosen (Table 2). The second pair of oligonucleotides (CfMVdiag-F/CfMVdiag-R) was designed by the Primer3 software (http://bioinfo.ut.ee/primer3-0.4.0/) [22] based on complete genome sequence of Russian CfMV isolate (L40905). The primers permit the amplification of a fragment of CP coding sequence of CfMV (Table 2). The selected oligonucleotides were used with a Transcriptor One-step RT-PCR Kit (Roche, Mannheim, Germany). In order to determine the optimal conditions of duplex-RT-PCR assay the different combinations of primers concentration and different temperatures of annealing were used. The reactions contained 10 µL of 5 × RT-PCR Reaction Buffer, 1µL of Transcription Enzyme Mix, 2 µL of primer pairs (in various tested concentrations: 0.6 µM or 0.4 µM or 0.3 µM), 1 µL of isolated RNA and they were supplemented to a final volume of 50 µL with sterile water. Reverse transcription was performed at 50 °C for 30 min. The initial denaturation was performed at 94 °C for 7 min, followed by 40 cycles of denaturation at 94 °C for 10 s, annealing at different tested temperatures of 56, 58 and 60 °C for 30 s, elongation at 68 °C for 60 s, and a final elongation at 68 °C for 7 min. RT-PCR products were verified by electrophoretical separation in 1% agarose gel containing Midori Green DNA stain (Nippon Genetics Europe GmbH, Düren, Germany) with 100-bp DNA ladder (Novazym, Poznań, Poland). Moreover, additional RT-PCR tests with BMV-ML1 sample were performed to verify natural co-infection with studied viruses. Selected CfCP-F1/CfCP-R2 [14] and CfMV-F/CfMV-R [11] primer pairs amplify specific DNA product of 776 pb and 655 pb in size, respectively. They enable to obtain complete CP coding sequence of CfMV. The reactions were performed following authors recommendations. The specificity of generated RT-PCR products was confirmed by sequencing. Amplified DNA fragments were excised from the gel and purified using Wizard® SV Gel and PCR Clean-Up System (Promega, Madison, WI, USA) and subjected to sequencing by Sanger method in external company (Genomed S.A., Warsaw, Poland). The obtained nucleotide sequences were edited and compiled in BioEdit software [23] and then were compared with the others deposited in GenBank database by BlastN. In the last step, the sensitivity of duplex-RT-PCR and conventional RT-PCR assays was estimated and compared. For this purpose 1 µL of tenfold serial dilutions (from 4.5 × 10^2^ to 4.5 × 10^–5^ ng/µL of total RNA) of BMV-ML1, sample co-infected with BMV and CfMV, were used as template for reactions. The detection limit was verified by electrophoresis as described above.

**Table 1:** Description of virus isolates used in this study

Isolate name	Geographical origin	Host	Collection date	Accession No.
BMV-Sz	Szelejewo	*Triticale*	2013	MW581058
BMV-Sr	near Środa Wielkopolska	*Zea mays*	2013	MW581059
BMV-Sosn	Sośnicowice	*Poa annua*	2016	MW581061
BMV-ML1	Gorzyń	*Dactylis glomerata*	2014	MW581062
BMV-ML2	Poznań	*Hordeum murinum*	2015	MW581065
BMV-Choj	Chojno	*Bromus hordeaceus*	2018	MW581057
BMV-R	Szelejewo	Water from ditch surrounding fields	2017	MW581064
BMV-Ch1	Choryń	Water from ditch surrounding fields	2017	MW581067
BMV-DBS	Bielany	Water from ditch surrounding fields	2017	MW581066
BMV-B	near Kraków	*Triticum aestivum*	2020	MW568015
BMV-Kon	Konin	*Poa annua*	2020	MW568017
CfMV-P1	Sośnicowice	*Dactylis glomerata*	2016	KX880413

**Table 2:** Primers used in duplex-RT-PCR

Primer’s name	Primer sequence (5′-3′)	Product size (bp)	References
BMV2-F	CTATAGCAAAGCGCTTTCGT		
BMV2-R	CAAACGTAGGGCACACTAGGG	798	Trzmiel et al. [17]
CfMVdiag-F	GATGGAGCCAGTCTCTCGAC		
CfMVdiag-R	CTCCCCACACGTTTGAAGTC	390	This study

## Results and discussion

**Fig. 1:**
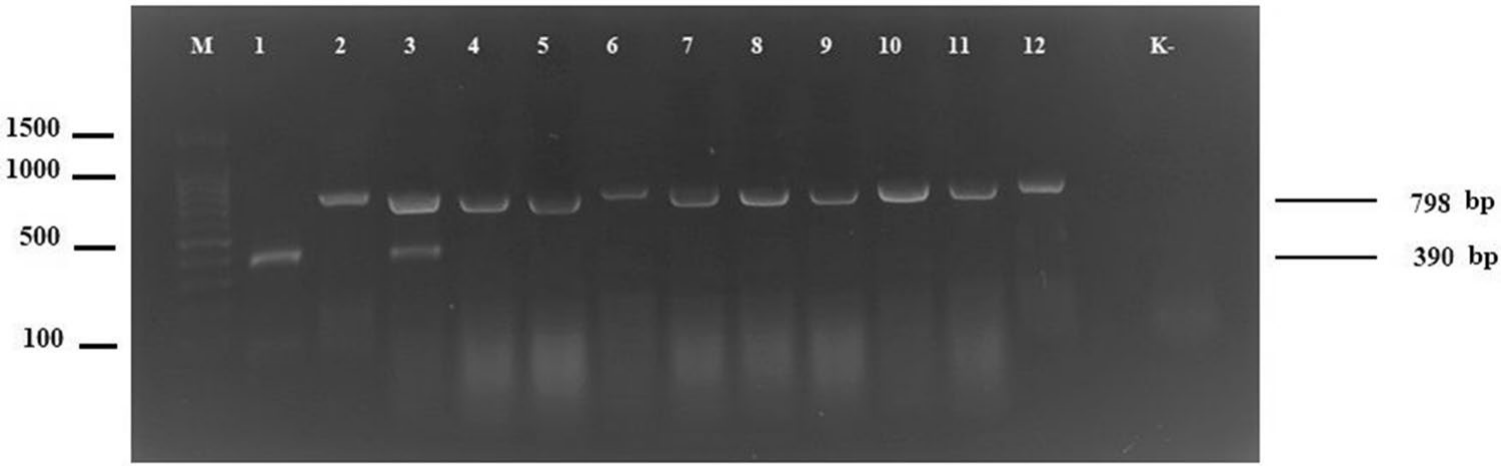
Analysis of duplex-RT-PCR products on agarose gel; lane M–100-bp DNA ladder (Novazym, Poznań, Poland), lanes: 1-CfMV-P1, 2-BMV-K, 3-BMV-ML1, 4-BMV-Sosn, 5-BMV-Sze, 6-BMV-ML2, 7-BMV-Choj, 8-BMV-Sr, 9-BMV-R, 10-BMV-DBS, 11-BMV-S, 12-BMV-Pk, lane K- negative control (total RNA from healthy barley)

**Fig. 2:**
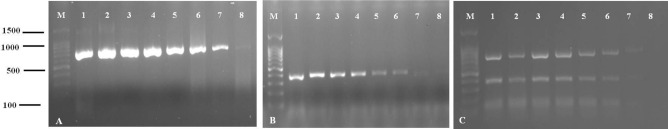
A comparison of sensitivity of conventional RT-PCR and duplex-RT-PCR techniques for BMV and CfMV detection. The figures present the electrophoretic separation of **a** RT-PCR BMV products, **b** RT-PCR CfMV products and **c** duplex-RT-PCR products. Fragments of 798 bp and 390 bp of BMV and CfMV were amplified from tenfold dilution of total RNA starting at 450 ng/µL. Lanes: M—100 bp DNA ladder (Novazym), 1–8—correspond to serial tenfold dilution of total RNA

Based on published results [2, 13] BMV and CfMV can be a new emerging threat for cereal crops. To date CfMV has been detected only in one locations in Poland [11] however, since first detection of BMV in 1999 in Poland, its presence has been reported in different regions of the country [5, 8]. Taking into account the possibility of further spread of the studied pathogens, the designing of specific diagnostic technique as the first step in the successful management of the diseases is needed. Existing diagnostic methods for BMV [2, 5, 8, 17–19] and CfMV [11, 13–16] have their limitations. ELISA is a time-consuming and laborious technique while conventional RT-PCR assay is able to detect only individual virus species in a single reaction. This limitations can be overcame using duplex-RT-PCR. In contrast to mentioned above methods, a newly presented here technique allows for the simultaneous detection and differentiation of BMV and CfMV in single reaction. In conclusion, this duplex-RT-PCR offers a significant time and cost-saving advantage. For the best amplification efficiency and specificity for both viruses, duplex-RT-PCR reactions were performed at different temperatures and the optimal annealing temperature was found to be 56 °C. Similarly, the analyses confirmed that the sensitivity of the reaction was correlated with the final concentration of the primers and balanced amplification for both pathogens was achieved when the primers concentrations were 0.3 µM for BMV and 0.6 µM for CfMV. The amplified products were obtained only for tested CfMV and BMV samples whereas no amplicons were produced for the samples of healthy barley and wheat plants. Additionally, for BMV-ML1 sample both specific RT-PCR products were obtained (Fig. 1). Sequencing of chosen duplex-RT-PCR products (798 bp for BMV and 390 bp for CfMV) confirmed their specificity. Comparative nucleotide sequences analyses showed high similarity of studied BMV and CfMV isolates and others deposited in GenBank database (over 99% identity). Moreover, the results of additional comparative analysis of complete CP nucleotide sequence of CfMV, amplified with BMV-ML1 sample, showed a 95% similarity with the corresponding fragments of the nucleotide sequences of CfMV-No (DQ680848), CfMV-Oxford (FJ669143); 94% with CfMV-Ohio (MF621330), CfMV-Russia (L40905), CfMV-Japan (AB040447) and only 93% with CfMV-P1 (KX880413). The results confirm the distinctiveness of the “newly detected” CfMV-ML1 isolate. Complete CP coding sequence of CfMV-ML1 (762 nt long) was deposited in the NCBI GenBank database (MW 147114). Developed here diagnostic system verifies the results obtained in the past and confirms the second location of CfMV in Poland with the presence of natural co-infection of BMV-ML1 and CfMV-ML1 in Wielkopolska region. This sample was used in next comparative analyses. Conventional RT-PCR reactions were able to detect BMV and CfMV in as little as 0.45 pg/µl and 4.5 pg/µl of total RNA, respectively (Fig. 2a, b). Duplex-RT-PCR was capable to detect viruses in as low as 4.5 pg/µl of total RNA (Fig. 2c). Although duplex-RT-PCR was tenfold less sensitive than conventional RT-PCR but it was still able to detect two viruses occurring in plant tissues in very low concentration. This technique allows for effective detection of co-infections with BMV and CfMV as early as 14 days after inoculation. Obtained results are comparable with those reported by other authors [21]. In conclusion, mentioned above results indicates that optimized in this study technique is useful diagnostic tool that can be routinely used by phytosanitary services for rapid identification of studied viral infection in cereals. To our knowledge, this is the first report of duplex-one-step-RT-PCR assay for detection of BMV and CfMV.

## Data Availability

The data of this study are available from the author upon request.
